# Spiders (Arachnida: Araneae) of PSU’s Botanical Garden (Perm, Russia)

**DOI:** 10.3897/BDJ.13.e163152

**Published:** 2025-09-23

**Authors:** Evgeniia Plakkhina, Artëm Sozontov, Sergei Esyunin, Natalya Ivanova, Dmitry Shumigay

**Affiliations:** 1 Perm State University, Perm, Russia Perm State University Perm Russia; 2 Institute of Plant and Animal Ecology (IPAE UB RAS), Ekaterinburg, Russia Institute of Plant and Animal Ecology (IPAE UB RAS) Ekaterinburg Russia; 3 Institute of Mathematical Problems of Biology RAS – the Branch of Keldysh Institute of Applied Mathematics of Russian Academy of Sciences, Pushchino, Russia Institute of Mathematical Problems of Biology RAS – the Branch of Keldysh Institute of Applied Mathematics of Russian Academy of Sciences Pushchino Russia

**Keywords:** Aranei, occurrence, long-term monitoring, biodiversity, greenhouse, urban fauna

## Abstract

**Background:**

The Botanical Garden of Perm State University was founded in 1916 and has a suitable site for studying urban invertebrate fauna. However, despite this, very few studies have been conducted on this topic over the past 100 years. Only a few individual works on greenhouse pests have been done. The first studies of the spider fauna on the territory of the botanical garden began in 2012. Studies of the seasonal activity of individual species, the composition of spider communities in uncultivated areas and alien species living in greenhouses have been conducted over the past 10 years.

**New information:**

This paper summarises primary field data from studies on the fauna of ground-dwelling spiders in open areas and greenhouses of PSU's Botanical Garden, collected in 2012 and during the period 2021–2024. The dataset "Spiders (Arachnida: Araneae) of PSU’s Botanical Garden (Perm, Russia)" describes the assemblage structure of spiders (list of species and their abundance), age-sex composition and seasonal dynamics. The dataset includes 714 events and 3468 corresponding occurrences. In total, 9088 specimens were collected, representing 114 species from 78 genera and 17 families. About 8400 individuals were identified to the species level. The dataset is complemented by a detailed description of the vegetation coverage at the study sites. This dataset considerably expands the Global Biodiversity Information Facility (GBIF) data on the distribution of spiders in the Urals. It provides new important information about urban fauna in general, as well as data on greenhouse and agrocenosis populations. Occurrences from both outdoor and indoor areas add a unique survey of microhabitats to the regional spider records available through GBIF.

## Introduction

The spiders of the Urals are a well-studied group (Esyunin 2015), but are still poorly represented on global digital biodiversity maps (Nesterkov et al. 2020, Vorobeichik et al. 2022, Sozontov et al. 2023, Mikhailov et al. 2024, Ukhova et al. 2024, GBIF.org 2025a, GBIF.org 2025b). In order to fill this gap, our research team is working on the digitisation of scientific biodiversity maps of the Ural spiders (Plakkhina et al. 2024, Sozontov and Mikhailov 2024). During this project, over 30,000 occurrences of spiders were digitised. These results have shown that the literature data on the synanthropic fauna of the Urals is extremely limited. Therefore, our field data of spider diversity collected in Perm can considerably expand the information in digitised literature data.

The fauna of arachnids in the Perm Krai has been well studied, but very few works have been conducted in Perm City. These publications either do not provide sufficient information on occurrences (Kuntsevich 2019), the lists of species are not detailed (Shumilovskikh 2001) or only the findings of synanthropic species are reported (Esyunin 1995). Some information about the local spider species can also be found in the "Definitive Guide to Invertebrate Animals of the City of Perm" (Aleksevnina et al. 2014) and in "Catalogue of the Spiders (Arachnida: Aranei) of the Urals" (Esyunin and Efimik 1996).

The Botanical Garden of Perm State University (PSU) was established in 1916. Despite providing a suitable location for studying the urban invertebrate fauna, no such research has been conducted over the past 100 years, with the exception of occasional studies on greenhouse pests (Chashchukhina 2007).

**Figure 1.**
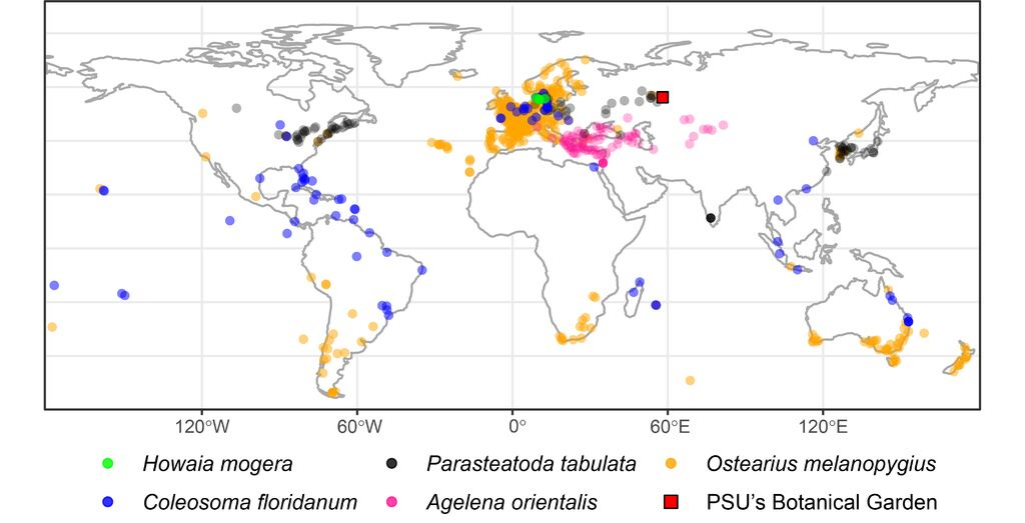
Map showing the ranges of introduced spider species collected at PSU's Botanical Garden (red square – Perm, the collection point), based on data from GBIF.org (2025c).

The spider fauna of the PSU Botanical Garden has been studied since 2012 (Plakkhina 2013, Sokolova and Plakkhina 2013). The first data on the composition of the ground-dwelling spider community were collected as part of a study on uncultivated vegetation areas in 2012 (Esyunin and Plakkhina 2022). The individuals of the alien species *Howaia mogera* (Yaginuma, 1972) were discovered within the greenhouse complex in 2017 (Esyunin et al. 2019). This was the reason to make a closer examination of the spider fauna in the botanical garden. Trap lines were installed in greenhouses and outdoor areas in 2021. The data collected during this period indicated that the examination of particular areas of the PSU's Botanical Garden, which vary in terms of floral composition, could be interesting from a faunistical and ecological perspective. The findings of the introduced spider species are based both on the results of a previous study in 2021 and on extensive sampling in 2022 using Merike traps. Their presence is linked to the importation of plants and highlights the role of heated greenhouses as entry points for alien invertebrates in the region. Despite being primarily confined to the heated greenhouses, isolated individuals were found outdoors: *H. mogera* (1 male, 2 females) and *Ostearius melanopygius* (8 males, 3 females). While *H. mogera* is unlikely to establish outdoors due to its subtropical affinity, *O. melanopygius* shows greater potential for local persistence, as evidenced by its synanthropic success and broader tolerance to temperature variations (Rozvałka et al. 2013). The global distribution ranges of the introduced spider species recorded at the collection site are shown in Fig. 1.

The dataset (Plakkhina et al. 2025) presents the results of long-term research on the arachnid fauna in PSU's Botanical Garden. During the study period (2012 and 2021–2024), 9088 individuals were recorded (114 species from 78 genera and 17 families). About 8400 individuals have been identified to the species level. The largest species diversity was found in the families Linyphiidae (55 species, 38% of individuals), Lycosidae (14 species, 41% of individuals), Theridiidae (13 species) and Gnaphosidae (6 species).

The dataset includes occurrences of spider species new to the Perm Region and alien to Russia, which were mentioned in ecological (Plakkhina and Esyunin 2022) and faunistic articles (Esyunin et al. 2024). All records are publicly available and respond to the Darwin Core data standard (Wieczorek et al. 2012). The dataset is complemented by a detailed description of the vegetation coverage on the study areas.

## General description

### Purpose

Arachnological research is a component of the scientific investigation into the structure of arthropods communities inhabiting the soil surface and litter within the PSU's Botanical Garden. The aim of the project is to conduct a comprehensive survey of the biodiversity and long-term monitoring of terrestrial invertebrates including ground beetles (Carabidae), weevils (Curculinoidea), millipedes (Myriapoda), harvestmen (Opiliones) and other taxa. The purpose of this article is to compile all available data on spiders that inhabit open areas and the greenhouses of the Botanical Garden and to create a current list of species and information on their occurrence.

## Project description

### Title

Terrestrial invertebrates of the PSU’s Botanical Garden (Perm)

### Personnel

Plakkhina E.V., Esyunin S.L.

### Study area description

Perm is a large industrial city, with more than a hundred companies operating in various sectors of the economy, such as mechanical engineering, fuel and energy complex and chemical and woodworking industries (Buzmakov et al. 2011). It is located on the edge of the East European Platform (Pre-Ural Region), in the central part of the Perm Krai (Voronov 2010). It is situated at the confluence of the Chusovaya and Sylva Rivers into the Kama River and extends along its banks for 65 kilometres. The city has an area of over 80,000 hectares (Voronov 2016). The city's territory is situated from 15 to 160 m a.s.l. (above sea level); some parts reach 250 m. Perm is located in a gently undulating, elevated valley, which is cut through by the Kama River and its tributaries (Voronov 2010).

Perm's climate is temperate continental: the warmest month is July (+17.9°C), the coldest month is January (–14.7°C). During the year, west and southwest winds are prevalent (Shklyaev and Shklyaeva 2006). Snow lasts for an average of 161–162 days annually. The stable snow cover usually forms at the end of October or the beginning of November. A thickness of the snow cover reaches several tens of centimetres (Buzmakov et al. 2011).

Soddy-podzolic soils are the most prevalent in Perm, with heavy-loam and clay textures dominating. Sandy and sandy-loam soils are also present, while specific technogenic soil types occur in squares and lawns. There are no natural soils on the territory of the Botanical Garden. During winter, soils can freeze to a depth of up to 80 centimetres (Voronov 2010).

Perm is located in a zone of southern taiga, characterised by spruce-fir forests mixed with linden and other deciduous trees; however, a significant portion of these forests has been cleared for residential development and garden plots established on the land (Voronov 2010) and a specific type of vegetation has emerged in urban areas. This vegetation is created and maintained artificially from both the local and introduced flora (Buzmakov et al. 2011).

### Design description

**Figure 2.**
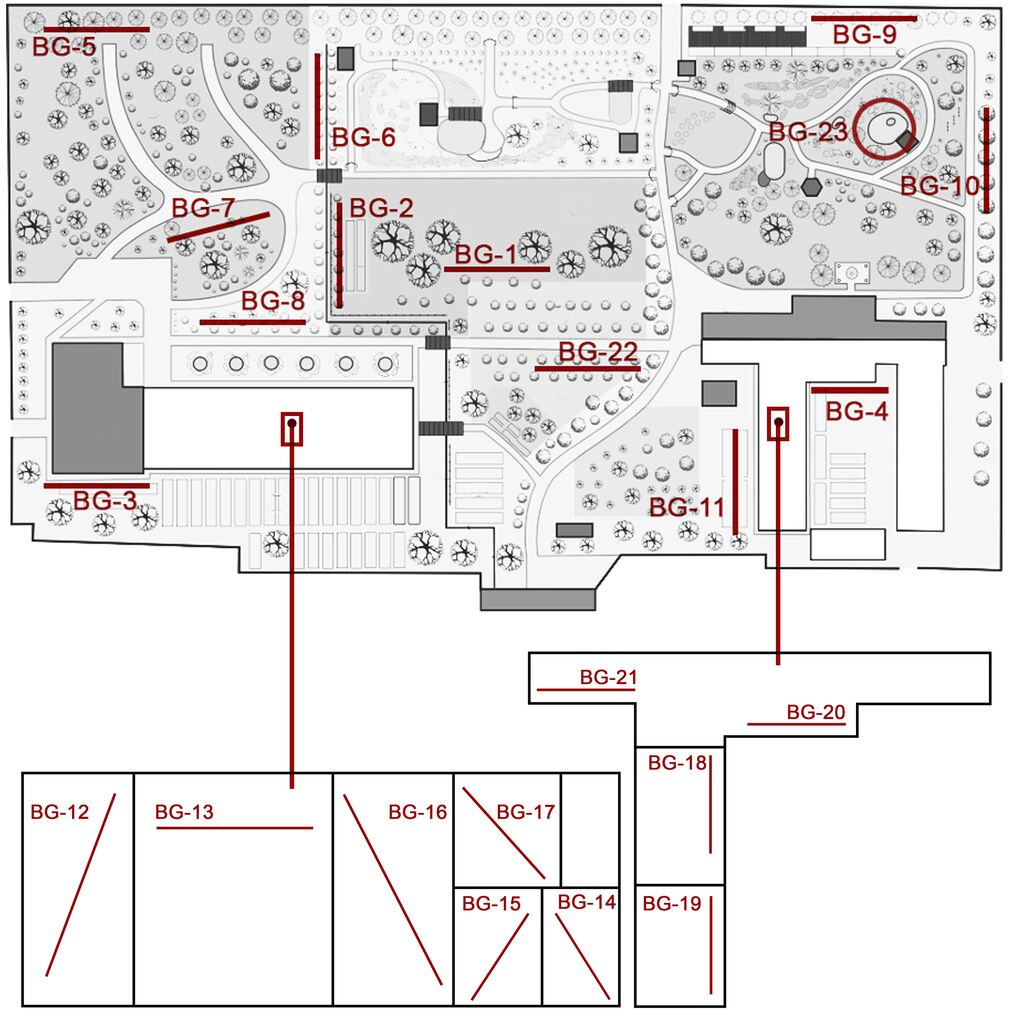
Plot locations on the map of PSU's Botanical Garden.

The data were derived from complex research on invertebrates inhabiting the soil surface and litter, which were carried out at 23 sampling plots (Fig. 2). Each plot has a brief description and information about the vegetation (see below). All records are georeferenced and contain metadata such as date, altitude, location, collecting method and sampling effort, which allows them to be used in quantitative environmental studies. Weather data are obtained from the records of the PSU's meteorological station. These data are not included in the dataset, but are available available upon request.

**Monitoring plots**

**Figure 3.**
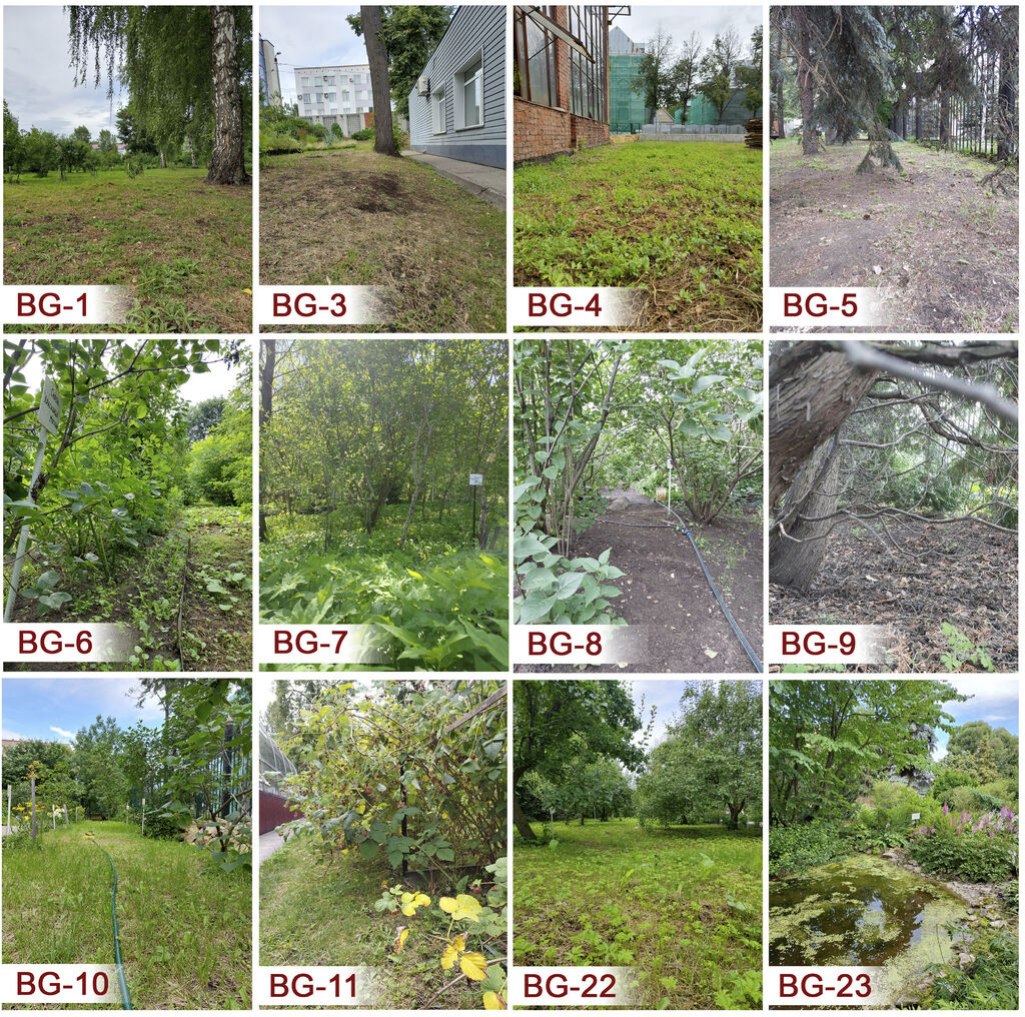
Some open-air plots of PSU's Botanical Garden.

**Figure 4.**
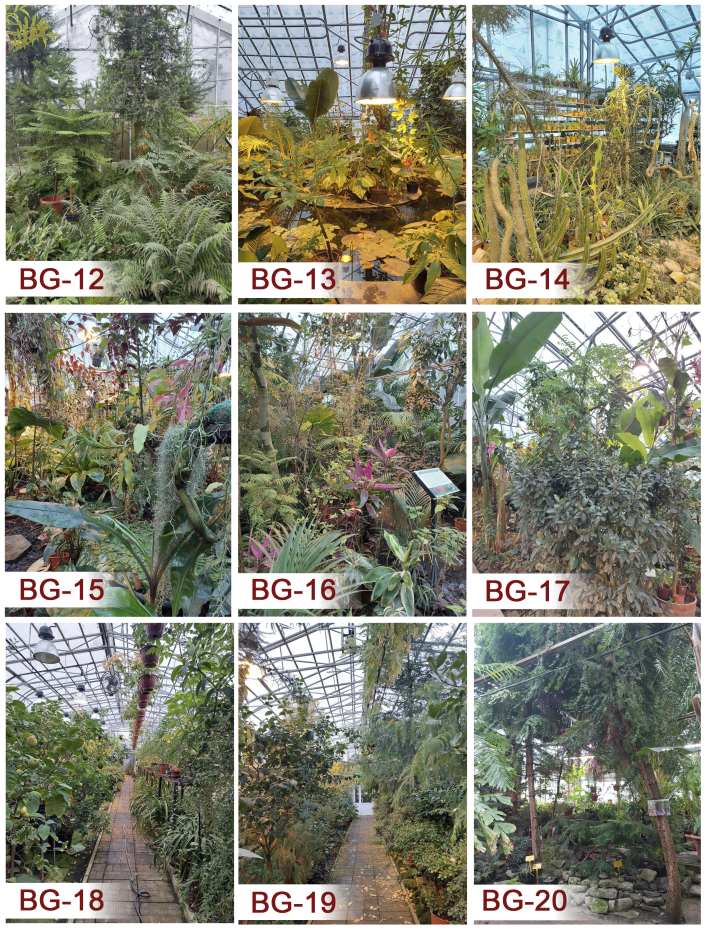
Some plots inside the greenhouse complex of the PSU's Botanical Garden.

Monitoring of the terrestrial invertebrate communities was carried out at 13 open-air plots and 10 compartments of the greenhouse complex. For open-air plots (Fig. 3), the following characteristics are presented: the presence of trees, the density of vegetation, the type of soil, the sun exposure and details of human activity. Key environmental parameters at each sampling site – including sun exposure, soil type and herb layer projective density/cover – were assessed by visual estimation. Sun exposure was estimated by observed canopy openness (%) and shadow patterns. Herb layer density/cover was assessed visually. Soil type was determined through manual texture analysis. This approach does not provide quantitative metrics, but produces descriptors of site conditions, sufficient enough for the aims of this study. The dataset is complemented by a detailed description of the vegetation coverage at the study sites (Suppl. material 1), as well as basic climatic conditions (Table 1) and a brief description of plant compositions for the plots in the greenhouse complex (Fig. 4). The vegetation data are included to facilitate future ecological analyses, specifically to examine the relationships between plant community composition, habitat structure and spider assemblage dynamics.

**BG-1 – Lawn under birch trees**

The plot with natural herbage under a row of four mature birch trees in a well-lit place. During the entire vegetation season, the herb layer on this plot was thin and sparse. Sun exposure is medium. The soil is a mixture of sand and peat. No special maintenance is performed on the plot.

**BG-2 – Vegetation along heat transfer pipes**

The plot of ruderal vegetation is located along the main line of the campus heat supply system, which ensures earlier snowmelt and better warming of the ground surface in the spring and autumn. There are no trees on the plot. The herb cover is 100% and reaches a height of 50–60 cm in summer. The soil is a mixture of sand and peat. Sun exposure is medium. No special maintenance is performed on the plot.

**BG-3 – Lawn behind the greenhouses**

The lawn about 1.5 m wide is located between the greenhouse and the flowerbeds where seedlings of shrubs and trees are grown. Several mature linden trees are growing on the lawn. Lawn grass cover of about 100% with occasional individuals of weeds and other ruderal plants. The soil is a mixture of sand and peat. The density of the soil is medium. Sun exposure is high. The grass on the plot is regularly mowed and watered via automatic sprinklers.

**BG-4 – Ruderal vegetation**

The plot is located behind the memorial greenhouse. There are no trees on the plot. The herbage is dense, but not high. The soil on the plot is dense, rocky with buried construction debris. Sun exposure is high. The grass is mowed in late May, mid-July and the last decade of August.

**BG-5 – Spruce trees plantings**

The plot is located in the mature spruce plantings with dense canopies. The herbage is sparse, consisting mostly of ruderal species entering from the campus lawns. The soil is a mixture of sand and peat. The density of the soil is medium. Sun exposure is medium. No special maintenance is performed on the plot.

**BG-6 – Phlox plantings**

The cultivated area located along the main line of the campus heat supply system. There are no trees, but a few young lilac shrubs are growing on the plot. The herbage is represented by phlox plantings, which are high, but not very dense. The soil is sandy. Sun exposure is medium. The site is regularly maintained and kept free of weeds.

**BG-7 – Arboretum**

The plot with plantings of various deciduous tree species. Herbaceous layer coverage exceeds 85%. The soil is peat. Sun exposure is low. The herbs are mowed twice during the summer.

**BG-8 – Lilac bush plantings**

The cultivated area with various lilac cultivars. Several low apricot trees also grow on the plot. Herbaceous layer is sparse. The soil is sandy. Sun exposure is low. No special maintenance is performed on the plot.

**BG-9 – Thuja tree plantings**

The thuja plantings are located along the fence of the botanical garden. The soil is sandy, covered with a 4-cm layer of thuya litter. Herbaceous cover is nearly absent; even ruderal species entering from campus lawns are rare. Sun exposure is low. No special maintenance is performed on the plot.

**BG-10 – Lawn near the fence**

The lawn is located along the fence of the Botanical Garden, approximately 1.5 m wide. Several individuals of young lilacs are growing on the plot. The herbage on the plot formed by dense grass with some ruderal species. The soil is a mixture of sand and peat. Sun exposure is high. The grass on the plot is regularly mowed and watered via automatic sprinklers.

**BG-11 – Raspberry plantings**

The plot without trees, with plantings of raspberry. The soil is peat. During early summer, raspberry bushes are small, but form dense cover by mid-summer. Sun exposure is low. The plot is regularly weeded, loosened and watered.

**BG-22 – Fruit trees**

The plot with fruit (apple and pear) trees. Grass and *Aegopodium podagraria* dominate the herbaceous layer; weeds and ruderal plants are rare; the cover of the layer is about 85%. The soil is a mixture of sand and peat. The density of the soil is medium. Sun exposure is medium. The herbs on the plot are regularly mowed and watered with automatic sprinklers.

**BG-23 – Pond**

On the banks of the pond there is an artificial plant community consisting of trees (cedar and spruce), bushes (willow and juniper) and herbaceous plants (*Aegopodium podagraria* and *Hosta hybrida*). The soil is peat with scattered stones (alpine rock features). Sun exposure is low. The plot is regularly weeded, loosened and watered.

Vegetation cover surveys were carried out at the following sites: from BG-3 to BG-11, BG-22 and BG-23. All studied plots are characterised by species common to disturbed habitats and roadsides. Most of the species are typical to the flora of the Perm Region and a small number of them are introduced to the botanical garden. All plots contain perennial, annual and biennial plant species. Some species are recorded only at the beginning of the growing season (ephemeroids, whose shoots die off by mid-summer). Others appear between mid-summer and autumn. Almost all woody plants and shrubs are introduced, planted in permanent exhibitions over different years and vary in age. All these facts lead to heterogeneous composition of the plots and their changes during the snowless period.

The indoor expositions are located in the stock greenhouse with an area of 1080 m^2^. The collections, numbering more than 2200 plant species, represent typical natural communities of the Tropics and Subtropics. The plots from **BG-12** to **BG-17** are located in a new part of the greenhouse complex, which was built in 2010; the plots from **BG-18** to **BG-21** are in the memorial part of the greenhouse complex, which was built in 1930. The descriptions of the **BG-12–BG-16** plots are presented according to the monograph of Shumikhin (2015). Plot BG-17, although situated in the same new section (built 2010), was not described in this monograph because this specific section was still under development or not fully accessible at the time of his work. The description of BG-17 provided in this study, as well as those for the plots in the memorial section (BG-18 - BG-21), are based on our own field assessment.

**BG-12 – Permian period**

The greenhouse compartment with an area of 214.24 m² with the exhibition "Plants of the Permian Geological Period", which is formed from living samples of systematic groups of plants from that period.

**BG-13 – Wet tropics**

The exposition with an area of 321.34 m² is an imitation of a tropical rainforest with appropriate microclimatic features. The plants of the humid tropical forests of the palaeotropical, neotropical and Australian floristic kingdom are presented. The compartment features reservoirs that support typical aquatic and coastal vegetation.

**BG-14 – Cacti and succulents**

The exhibition, designed as a rocky "Mexican" desert landscape, covers an area of 81 m². A group of plants from arid habitats is represented here. Planting is sparse because succulents need a lot of space around them for growth.

**BG-15 – Epiphytes**

The 79.33 m² exposition features plants from the families Araceae, Bromeliaceae, Orchidaceae and Piperaceae. Epiphytic, insectivorous and myrmecophilic traits are demonstrated here. Most plants in this group need certain conditions: high humidity and high temperature.

**BG-16 – Dry tropics**

The exposition covers an area of 213.77 m². Dry tropical regions are characterised by a change of rainy and dry seasons; therefore, the compartment has two maintenance modes: summer (humid and hot) and winter (drier and cooler). The exposition is divided into zones representing palaeotropics and neotropics, including Australia. The plantings in this area are less dense than those in **BG-13**.

**BG-17 – Useful tropical plants**

The exhibition "Useful Tropical Plants" demonstrates plants used by humans for practical purposes. Due to the specific conditions required for their maintenance, this section is similar to the **BG-13**, featuring high humidity and consistently high temperatures.

Plots **BG-18 – Orchids, BG-19 – Subtropics, BG-20 – Old greenhouse and BG-21 – Old greenhouse, cold** are located in a large Subtropical Plant Exhibition, which is characterised by a growing season during the summer and a dormant period during the winter, with temperature and humidity levels similar to natural conditions. The exhibition showcases a variety of plants including those from the Rutaceae, Myrtaceae, Ericaceae, Orchidaceae and Theaceae families, as well as tropical fruit trees and representatives of the Australian flora. In **BG-18**, traps were placed amongst the roots of orchids. In **BG-19**, traps were placed under citrus trees. In **BG -2**, traps were placed along the line of the heat supply system. In **BG -21**, traps were placed in the cooler part of this section, near the entrance.

### Funding

The research was supported by grant # 24-24-00460 from the Russian Science Foundation.

## Sampling methods

### Sampling description

All data on ground-dwelling spiders (except for events in greenhouses in 2022) were collected using pitfall traps and plastic cups (75 mm in diameter) filled with 4% formaldehyde. The traps were placed in rows: of 10 in open areas and of five in the greenhouse compartments. The traps were set 2 m apart. The sampling duration typically ranged from one to two weeks, with periods varying from 4 days to 3 weeks.

Within the greenhouse compartments during 2022, spiders were collected using modified Möricke traps, yellow-foamed polystyrene food containers filled with a 1% formaldehyde preservative solution (+ detergent to reduce surface tension). The traps were placed on the substrate along the same trap lines as in 2021. To avoid overlap with watering schedules, the events were held from Friday evening until Monday morning, every two weeks.

### Quality control

The initial identification of species was conducted by S.L. Esyunin, all subsequent identifications being carried out by E.V. Plakkhina according to identification keys in "Spiders of Europe" (Nentwig et al. 2024). Juvenile individuals were identified up to the species, genus or family level, depending on the informative value of morphological features. Taxonomic nomenclature follows the World Spider Catalogue (World Spider Catalog 2025).

### Step description

The project is long-term and continues.

## Geographic coverage

### Description

**Figure 5.**
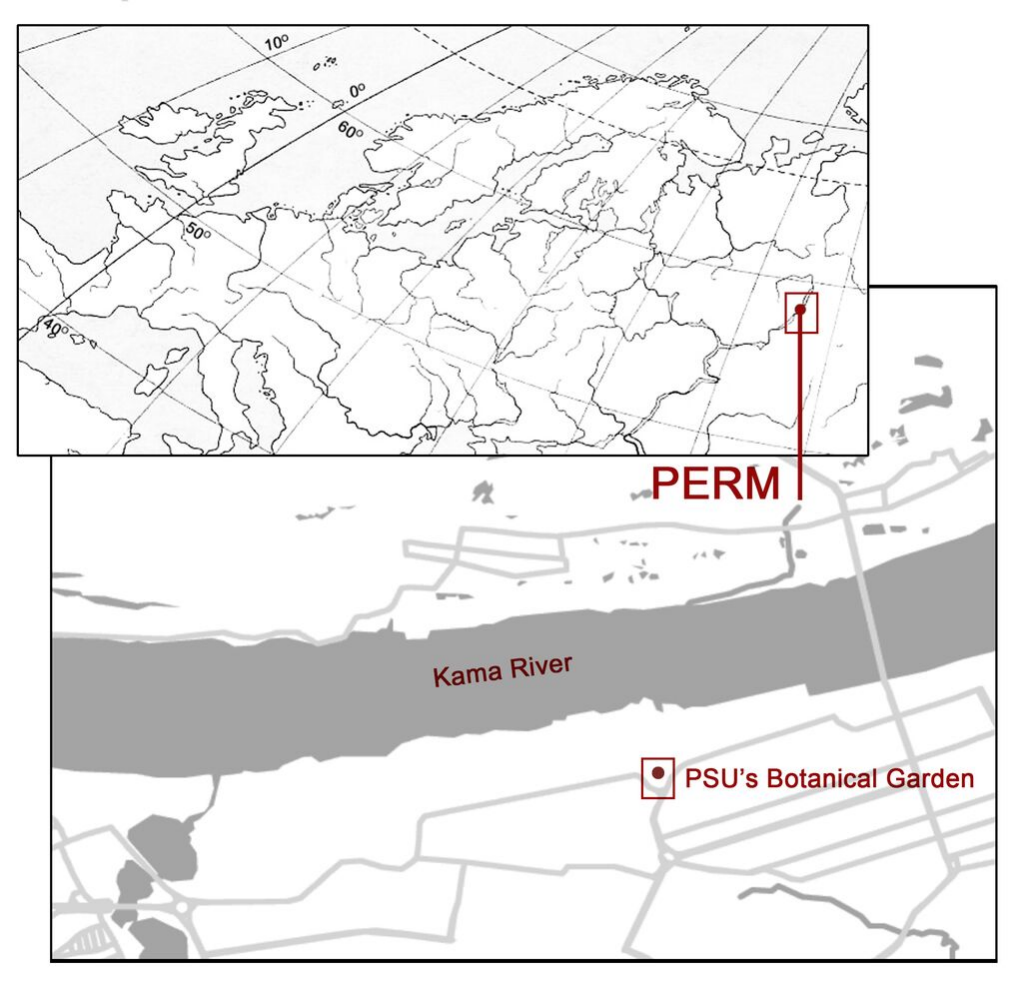
The location of PSU's Botanical Garden.

The Botanical Garden is situated within the city boundaries, on the territory of the PSU's campus (Fig. 5). It covers an area of approximately 1.97 hectares (Shumikhin 2015) and is surrounded on three sides by urban areas and, on the fourth side, by the Trans-Siberian railway line. The Botanical Garden is located within the vicinity (about 800 m in a straight line) of a major river, the Kama. More than 5,000 different plant species are cultivated here, represented by over 7,500 different varieties (Shumikhin 2015). The territory is divided into exhibition, production and scientific zones. All these features make its landscapes interesting and convenient site for observing changes in the structure of invertebrate communities, including spiders, during the entire snowless period of the year.

### Coordinates

58.005 and 58.007 Latitude; 56.188 and 56.187 Longitude.

## Taxonomic coverage

### Description

**Figure 6.**
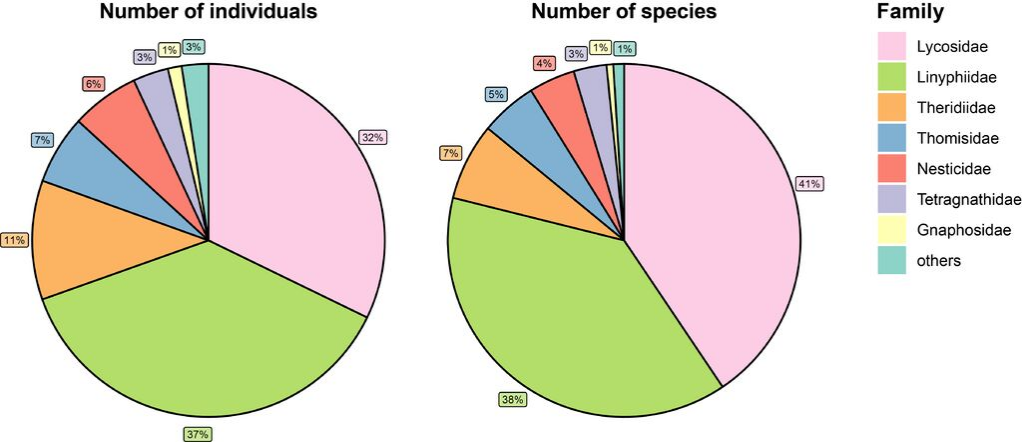
The proportion of the abundance (left) and the number of species (right) of spiders within each family in surveys on the territory of PSU's Botanical Garden.

The dataset includes 3,469 records of spider (Arachnida: Araneae) occurrences, representing 114 species from 78 genera. The proportion of abundance and species richness within each family in the surveys conducted on the territory of the PSU's Botanical Garden is shown in Fig. 6.

### Taxa included

**Table:** 

Rank	Scientific Name	Common Name
order	Araneae	Spiders

## Traits coverage

The listed species are characterised by their range type and preferred vegetation layer (Suppl. material 2). The "Range group (range type)" column shows the group and type (in brackets) of the longitudinal range and the "Range zonal" column shows the groups and type (in brackets) of the latitudinal range. The preferred vegetation layer is given according to monograph by Sozontov and Esyunin (2022) and may include one or more of the following: litter, moss cover, soil surface, herb layer, shrub layer, tree trunks and canopy.

## Temporal coverage

**Data range:** 2012-4-13 – 2012-10-20; 2020-12-30 – 2021-12-24; 2022-4-26 – 2022-11-11; 2023-3-31 – 2023-11-10; 2024-4-12 – 2024-12-06.

## Usage licence

### Usage licence

Creative Commons Public Domain Waiver (CC-Zero)

### IP rights notes

This work is licensed under a Creative Commons Attribution (CC-BY 4.0) License.

## Data resources

### Data package title

Spiders (Arachnida: Araneae) of PSU’s Botanical Garden (Perm, Russia)

### Resource link

https://doi.org/10.15468/3hztr6

### Alternative identifiers

https://www.gbif.org/dataset/ea20b36b-a397-4e46-b1f1-a7a409405371

### Number of data sets

1

### Data set 1.

#### Data set name

Spiders (Arachnida: Araneae) of PSU’s Botanical Garden (Perm, Russia)

#### Data format

Darwin Core

#### Download URL

http://gbif.ru:8080/ipt/resource?r=psu_botgar_spiders

#### Description

The dataset is based on a complex study of the ground-dwelling invertebrates at the Botanical Garden of Perm State University. The study was conducted using pitfall-traps at 23 sampling sites between 2012 and 2024. Ten plots were located in greenhouses, while thirteen were located outdoors. The dataset includes 3,468 records of spiders’ (Arachnida: Araneae) occurrences, representing 114 species from 78 genera and 9088 individuals. All records are georeferenced and linked with essential metadata, such as the date, altitude and type of habitat, as well as information about the collection method and the amount of sampling effort. Research in the botanical garden's vicinity continues and the dataset will be expanded with further information.

**Data set 1.** 

Column label	Column description
parentEventID	An identifier for the broader dwc:Event that groups this and potentially other dwc:Events. A variable.
eventID	An identifier for the set of information associated with a dwc:Event (something that occurs at a place and time). May be a global unique identifier or an identifier specific to the dataset. A variable.
eventDate	The date-time or interval during which a dwc:Event occurred. For occurrences, this is the date-time when the dwc:Event was recorded. Not suitable for a time in a geological context. A variable.
samplingProtocol	The names of, references to, or descriptions of the methods or protocols used during a dwc:Event. A variable.
sampleSizeValue	A numeric value for a measurement of the size (time duration, length, area or volume) of a sample in a sampling dwc:Event. A variable.
sampleSizeUnit	The unit of measurement of the size (time duration, length, area, or volume) of a sample in a sampling dwc:Event. A constant ("trap-days").
samplingEffort	The amount of effort expended during a dwc:Event. A variable.
habitat	A category or description of the habitat in which the dwc:Event occurred. A variable.
locationID	An identifier for the set of dcterms:Location information. May be a global unique identifier or an identifier specific to the dataset. A variable.
higherGeography	An identifier for the geographic region within which the dcterms:Location occurred. A constant ("Europe | Russian Federation | Perm Krai| Perm").
continent	The name of the continent in which the dcterms:Location occurs. A constant ("Europe").
country	The name of the country or major administrative unit in which the dcterms:Location occurs. A constant ("Russian Federation").
countryCode	The standard code for the country in which the dcterms:Location occurs. A constant ("RU").
stateProvince	The name of the next smaller administrative region than country (state, province, canton, department, region etc.) in which the dcterms:Location occurs. A constant ("Perm Krai").
county	The full, unabbreviated name of the next smaller administrative region than stateProvince (county, shire, department etc.) in which the dcterms:Location occurs. A constant ("Permskiy District").
municipality	The full, unabbreviated name of the next smaller administrative region than county (city, municipality etc.) in which the dcterms:Location occurs. Do not use this term for a nearby named place that does not contain the actual dcterms:Location. A constant ("Perm").
locality	The specific description of the place. A constant ("PSU's Botanical Garden").
minimumElevationInMeters	The lower limit of the range of elevation (altitude, usually above sea level), in metres. A constant ("105").
maximumElevationInMeters	The upper limit of the range of elevation (altitude, usually above sea level), in metres. A constant ("105").
decimalLatitude	The geographic latitude (in decimal degrees, using the spatial reference system given in dwc:geodeticDatum) of the geographic centre of a dcterms:Location. Positive values are north of the Equator, negative values are south of it. Legal values lie between -90 and 90, inclusive. A variable.
decimalLongitude	The geographic longitude (in decimal degrees, using the spatial reference system given in dwc:geodeticDatum) of the geographic centre of a dcterms:Location. Positive values are east of the Greenwich Meridian, negative values are west of it. Legal values lie between -180 and 180, inclusive. A variable.
geodeticDatum	The ellipsoid, geodetic datum or spatial reference system (SRS), upon which the geographic coordinates given in dwc:decimalLatitude and dwc:decimalLongitude are based. A constant ("WGS84").
coordinatePrecision	A decimal representation of the precision of the coordinates given in the dwc:decimalLatitude and dwc:decimalLongitude. A constant ("0.00001").
coordinateUncertaintyInMeters	The horizontal distance (in metres) from the given dwc:decimalLatitude and dwc:decimalLongitude describing the smallest circle containing the whole of the dcterms:Location. Leave the value empty if the uncertainty is unknown, cannot be estimated or is not applicable (because there are no coordinates). Zero is not a valid value for this term. A constant ("25").
georeferencedBy	A list (concatenated and separated) of names of people, groups or organisations who determined the georeference (spatial representation) for the dcterms:Location. A constant ("Plakkhina Evgeniia").
georeferencedDate	The date on which the dcterms:Location was georeferenced. A constant ("2024").
occurrenceID	An identifier for the dwc:Occurrence (as opposed to a particular digital record of the dwc:Occurrence).
basisOfRecord	The specific nature of the data record. A constant ("HumanObservation").
individualCount	The number of individuals present at the time of the dwc:Occurrence. A variable.
organismQuantity	A number or enumeration value for the quantity of dwc:Organisms. A variable.
organismQuantityType	A number or enumeration value for the quantity of dwc:Organisms. A constant ("individuals").
occurrenceStatus	A statement about the presence or absence of a dwc:Taxon at a dcterms:Location. A constant ("present").
scientificName	The full scientific name, with authorship and date information if known. When forming part of a dwc:Identification, this should be the name in the lowest level taxonomic rank that can be determined. This term should not contain identification qualifications, which should instead be supplied in the dwc:identificationQualifier term. A variable.
kingdom	The full scientific name of the kingdom in which the dwc:Taxon is classified. A constant ("Animalia").
phylum	The full scientific name of the phylum or division in which the dwc:Taxon is classified. A constant ("Arthropoda").
class	The full scientific name of the class in which the dwc:Taxon is classified. A constant ("Arachnida").
order	The full scientific name of the order in which the dwc:Taxon is classified. A constant ("Araneae").
family	The full scientific name of the family in which the dwc:Taxon is classified. A variable.
genus	The full scientific name of the genus in which the dwc:Taxon is classified. A variable.
specificEpithet	The name of the first or species epithet of the dwc:scientificName. A variable.
taxonRank	The taxonomic rank of the most specific name in the dwc:scientificName. A variable.
sex	The sex of the biological individual(s) represented in the dwc:Occurrence. A variable.
lifeStage	The age class or life stage of the dwc:Organism(s) at the time the dwc:Occurrence was recorded. A variable.
recordedBy	A person, group or organisation responsible for recording the originaldwc:Occurrence. A constant (“Plakkhina E.V.”).
identifiedBy	A list (concatenated and separated) of the globally unique identifier for the person,people, groups or organisations responsible for assigning the dwc:Taxon to the subject. A variable.
establishmentMeans	Statement about whether a dwc:Organism has been introduced to a given place and time through the direct or indirect activity of modern humans.

## Supplementary Material

0908E322-185C-5CFB-87F5-0C502EB845E2 10.3897/BDJ.13.e163152.suppl1 Supplementary material 1 List of plant species recorded Data type PDF Brief description List of plant species recorded in open-air plots. LF – life forms: H – herbs, S – shrubs, T – trees. File: oo_1393189.pdf https://binary.pensoft.net/file/1393189 Evgeniia Plakkhina, Artëm Sozontov, Sergei Esyunin, Natalya Ivanova, Dmitry Shumigay

802FAC32-D205-5643-9C4C-DEBD0BCB2B89 10.3897/BDJ.13.e163152.suppl2 Supplementary material 2 List of spider species Data type PDF Brief description List of spider species from PSU's Botanical Garden. File: oo_1393190.pdf https://binary.pensoft.net/file/1393190 Evgeniia Plakkhina, Artëm Sozontov, Sergei Esyunin, Natalya Ivanova, Dmitry Shumigay

## Figures and Tables

**Table 1.** Basic climatic conditions in the greenhouse complex of PSU's Botanical Garden.

Compartment	Relative humidity	Average air temperature (°C)			
		Winter	Spring	Summer	Autumn
**BG-12**	80–90	22.3	21.5	23.3	19.3
**BG-13**	90–100	28.6	23.6	23.2	23.2
**BG-14**	60–70	20.9	24.6	24.5	21.3
**BG-15**	95–100	24.1	23.4	23.3	21.7
**BG-16**	80	27.5	22.9	23.0	22.9
**BG-17**	80	26.0	24.4	23.2	22.0
**BG-18**	85–90	14.8	21.1	23.2	13.3
**BG-19**	85–90	15.1	19.7	23.1	13.8
**BG-20**	85–90	12.5	16.6	20.2	13.1
**BG-21**	85–90	11.6	17.7	21.0	12.5
