# Supplementary material for: Spiders (Arachnida: Araneae) of PSU’s Botanical Garden (Perm, Russia)
Source: Biodivers Data J. 2025 Sep 23;13:e163152. doi: 10.3897/BDJ.13.e163152 (PMC12485477; doi:10.3897/BDJ.13.e163152)
Supplement: Supplementary material 1 — List of plant species recorded [file bdj-13-e163152-s001.pdf]

Table 1. List of plant species recorded in open-air plots. LF – life forms: H – herbs, S – shrubs, T – trees.

LF	Family	Species	Number of open-air plots without prefix “BG”										
			**3**	**4**	**5**	**6**	**7**	**8**	**9**	**10**	**11**	**22**	**23**
H	Amaranthaceae	*Atriplex patula* L.			+	+			+				
H	Amaranthaceae	*Chenopodium album* Bosc ex Moq.	+		+	+	+	+	+				
H	Amaryllidaceae	*Allium caeruleum* Stschegl.							+	+			
H	Amaryllidaceae	*Allium chinense* G.Don											+
H	Apiaceae	*Aegopodium podagraria* L.		+	+	+	+		+	+	+	+	+
H	Apiaceae	*Conium maculatum* L.			+								
H	Apiaceae	*Heracleum sibiricum* L.		+									
H	Asparagaceae	*Ornithogalum umbellatum* L.				+	+	+				+	
H	Asparagaceae	*Hosta × hybrida* hort.											+
H	Asteraceae	*Achillea millefolium* L.	+	+	+								
H	Asteraceae	*Arctium tomentosum* Mill.	+							+	+		
H	Asteraceae	*Artemisia vulgaris* L.								+			
H	Asteraceae	*Bellis perennis* L.	+		+								
H	Asteraceae	*Cacalia tschonoskii* Koidz.											+
H	Asteraceae	*Cirsium arvense* (L.) Scop.		+	+				+	+			
H	Asteraceae	*Crepis tectorum* L.			+								
H	Asteraceae	*Erigeron canadensis* L.	+	+	+	+	+	+	+	+			+
H	Asteraceae	*Galinsoga quadriradiata* Ruiz & Pav.						+		+	+		
H	Asteraceae	*Hieracium aurantiacum* L.	+										
H	Asteraceae	*Lapsana communis* L.			+	+				+			
H	Asteraceae	*Leontodon autumnalis* L.			+					+			
H	Asteraceae	*Leucanthemum vulgare* Lam.			+								
H	Asteraceae	*Ligularia sachalinensis* Nakai											+
H	Asteraceae	*Matricaria chamomilla* L.				+							
H	Asteraceae	*Senecio vulgaris* L.			+				+	+	+		+
H	Asteraceae	*Sonchus oleraceus* L.	+	+		+	+	+	+	+	+	+	
H	Asteraceae	*Taraxacum officinale* Weber ex F.H.Wigg.	+	+	+	+	+	+	+	+	+	+	+
H	Asteraceae	*Tripleurospermum inodorum* (L.) Sch.-Bip		+	+			+	+	+	+		
H	Asteraceae	*Tussilago farfara* L.			+								
H	Balsaminaceae	*Impatiens parviflora* DC.							+				
S	Betulaceae	*Corylus avellana* L.					+						
H	Boraginaceae	*Myosotis arvensis* (L.) Hill								+			
H	Boraginaceae	*Myosotis sparsiflora* J.C.Mikan ex Pohl	+	+	+	+	+		+	+		+	+
H	Boraginaceae	*Symphytum officinale* L.		+		+						+	
H	Brassicaceae	*Capsella bursa-pastoris* (L.) Medik.	+	+	+	+	+	+	+	+	+		
H	Brassicaceae	*Erysimum cheiranthoides* L.			+				+	+			
H	Brassicaceae	*Rorippa sylvestris* (L.) Besser		+	+	+	+	+					
H	Brassicaceae	*Sisymbrium officinale* (L.) Scop.			+					+			
H	Brassicaceae	*Thlaspi arvense* L.				+				+		+	
H	Campanulaceae	*Campanula rotundifolia* L.											+
H	Campanulaceae	*Campanula urticifolia* F.W.Schmidt							+	+			+
S	Caprifoliaceae	*Lonicera tatarica* L.					+						
H	Caryophyllaceae	*Melandrium album* (Mill.) Garcke			+								

H	Caryophyllaceae	*Sagina procumbens* L.		+				+			+		
H	Caryophyllaceae	*Stellaria media* (L.) Vill.	+	+	+		+	+	+	+	+	+	
H	Convolvulaceae	*Convolvulus arvensis* L.		+	+	+						+	
S	Cornaceae	*Cornus alba* L.					+						
S	Cornaceae	*Cornus iberica* Woronow					+						
S	Cupressaceae	*Thuja occidentalis* L.							+				+
S	Ericaceae	*Rhododendron japonicum* (A.Gray) Suringar											+
H	Euphorbiaceae	*Euphorbia virgata* Waldst. & Kit.	+										
H	Fabaceae	*Medicago lupulina* L.	+										
H	Fabaceae	*Trifolium medium* L.	+	+	+		+				+		
H	Fabaceae	*Trifolium pratense* L.						+					
H	Fabaceae	*Trifolium repens* L.		+	+	+			+			+	
H	Fabaceae	*Vicia sepium* L.			+								
T	Fagaceae	*Quercus robur* L.			+					+			
T	Fagaceae	*Quercus rubra* L.								+			
H	Iridaceae	*Iris halophila* Pall.											+
H	Iridaceae	*Iris maackii* Maxim.											+
H	Iridaceae	*Iris uniflora* Pall. ex Link											+
T	Juglandaceae	*Juglans mandshurica* Maxim.								+			
T	Juglandaceae	*Juglans regia* L.					+						
T	Juglandaceae	*Pterocarya fraxinifolia* (Poir.) Spach					+						
H	Lamiaceae	*Glechoma hederacea* L.		+	+					+			
H	Lamiaceae	*Lamium album* L.		+	+				+	+	+		
H	Lamiaceae	*Lamium purpureum* L.		+	+		+	+		+			
H	Lamiaceae	*Leonurus quinquelobatus* Gilib.		+									
H	Lamiaceae	*Prunella vulgaris* L.										+	
H	Malvaceae	*Malva pusilla* Sm.			+			+		+			
T	Malvaceae	*Tilia × platyphylla* Gray					+						
T	Malvaceae	*Tilia cordata* Mill.	+										
T	Oleaceae	*Syringa vulgaris* L.				+		+		+		+	
H	Onagraceae	*Chamaenerion angustifolium* (L.) Scop.							+				
H	Onagraceae	*Epilobium palustre* L.			+	+			+		+		+
H	Oxalidaceae	*Oxalis stricta* L.	+	+		+		+					
H	Papaveraceae	*Chelidonium majus* L.	+	+	+			+	+	+			+
H	Phytolaccaceae	*Phytolacca americana* L.							+				
T	Pinaceae	*Abies sibirica* Ledeb.					+						
T	Pinaceae	*Picea pungens* Engelm.			+								
S	Pinaceae	*Pinus mugo* Turra											+
H	Plantaginaceae	*Plantago major* L.	+	+	+			+		+	+	+	
H	Plantaginaceae	*Veronica persica* Poir.		+			+						
H	Poaceae	*Agropyron repens* (L.) P.Beauv.		+						+	+		
H	Poaceae	*Agrostis tenuis* T.Bastard ex Roem. & Schult.		+						+			
H	Poaceae	*Bromus inermis* Leyss.		+									
H	Poaceae	*Dactylis glomerata* L.			+		+						
H	Poaceae	*Echinochloa crus-galli* (L.) P.Beauv.				+		+			+		
H	Poaceae	*Festuca pratensis* Schreb.			+								
H	Poaceae	*Poa annua* L.	+	+	+	+	+	+	+	+	+	+	+
H	Poaceae	*Poa pratensis* L.	+				+					+	
H	Polygonaceae	*Persicaria maculosa* Gray	+	+		+				+	+		+
H	Polygonaceae	*Polygonum aviculare* L.	+	+		+	+			+	+		

H	Polygonaceae	*Polygonum convolvulaceum* Lam.		+				+					
H	Polygonaceae	*Rumex acetosella* L.	+		+	+	+	+			+		
H	Polygonaceae	*Rumex confertus* Willd.	+	+	+	+	+	+		+	+	+	
H	Primulaceae	*Lysimachia nummularia* L.					+						
H	Ranunculaceae	*Anemone altaica* Fisch. ex C.A.Mey.										+	
H	Ranunculaceae	*Anemone canadensis* L.											+
H	Ranunculaceae	*Anemone ranunculoides* L.				+	+	+	+		+	+	
H	Ranunculaceae	*Aquilegia vulgaris* L.											+
H	Ranunculaceae	*Cimicifuga dahurica* (Turcz. ex Fisch. & C.A.Mey.) Maxim.											+
H	Ranunculaceae	*Ficaria verna* Huds.				+	+	+			+	+	
H	Ranunculaceae	*Ranunculus repens* L.		+			+						
H	Ranunculaceae	*Trollius chinensis* Bunge											+
S	Rhamnaceae	*Frangula alnus* Mill.					+						
S	Rhamnaceae	*Rhamnus cathartica* L.					+						
H	Rosaceae	*Alchemilla vulgaris* L.	+	+			+			+		+	
S	Rosaceae	*Crataegus chlorocarpa* Lenné & K.Koch					+						
H	Rosaceae	*Filipendula rubra* (Hill) B.L.Rob.											+
S	Rosaceae	*Kerria japonica* (L.) DC.											+
T	Rosaceae	*Malus domestica* (Suckow) Borkh.										+	
H	Rosaceae	*Potentilla anserina* L.	+	+	+	+	+	+	+	+			
T	Rosaceae	*Pyrus domestica* (L.) Ehrh.										+	
S	Rosaceae	*Rosa glauca* Pourr.					+						
S	Rosaceae	*Rosa sherardii* Davies			+								
S	Rosaceae	*Rubus idaeus* L.									+		
H	Rubiaceae	*Galium mollugo* L.	+		+	+				+			
T	Salicaceae	*Salix ledebouriana* Trautv.					+						
T	Salicaceae	*Salix pentandra* L.					+						
T	Sapindaceae	*Acer campestre* L.					+						
T	Sapindaceae	*Acer hyrcanum* Fisch. & C.A.Mey.					+						
T	Sapindaceae	*Acer platanoides* L.			+								
T	Sapindaceae	*Acer saccharinum* L.							+				
T	Sapindaceae	*Acer tataricum* L.					+						
H	Saxifragaceae	*Astilbe arendsii* Arends											+
H	Scrophulariaceae	*Verbascum thapsus* L.			+								
H	Solanaceae	*Hyoscyamus niger* L.			+	+							
H	Solanaceae	*Solanum dulcamara* L.					+	+					
H	Urticaceae	*Urtica dioica* L.	+	+	+	+	+	+	+	+	+	+	
T	Viburnaceae	*Viburnum opulus* L.					+						
S	Viburnaceae	*Viburnum sargentii* Koehne					+						
H	Violaceae	*Viola arvensis* Murray										+	
H	Violaceae	*Viola tricolor* L.				+		+	+				
