# Supplementary material for: Spiders (Arachnida: Araneae) of PSU’s Botanical Garden (Perm, Russia)
Source: Biodivers Data J. 2025 Sep 23;13:e163152. doi: 10.3897/BDJ.13.e163152 (PMC12485477; doi:10.3897/BDJ.13.e163152)
Supplement: Supplementary material 2 — List of spider species [file bdj-13-e163152-s002.pdf]

Table 3. List of spider species from PSU's Botanical Garden.

Family	Species	Range group (range type)	Range zonal	Stratum
Agelenidae	*Agelena labyrinthica* (Clerck, 1757)	Palaearctic (Trans-Palaearctic)	Subboreal	herb
Agelenidae	*Agelena orientalis* C.L.Koch, 1837	Ancient Mediterranean (SouthEuropean-CentralAsian)	Subboreal-semidesert	herb
Agelenidae	*Tegenaria domestica* (Clerck, 1757)	Cosmopolitan	Synanthropic	synanthrope
Agelenidae	*Tegenaria lapicidinarum* Spassky, 1934	Euro-Siberian (EastEuropean)	subboreal	ground, herbs
Araneidae	*Araneus diadematus* Clerck, 1757	Palaearctic (TransPalaearctic)	Temperate	shrub, canopy
Araneidae	*Singa hamata* (Clerck, 1757)	Palaearctic (Trans-Palaearctic)	Temperate	herb, shrub
Clubionidae	*Clubiona subsultans* Thorell, 1875	Palaearctic (Trans-Palaearctic)	Temperate	tree stems
Dictynidae	*Dictyna arundinacea* (Linnaeus, 1758)	Holarctic (Circum-Holarctic)	Polyzonal	herb, shrub
Dictynidae	*Dictyna uncinata* Thorell, 1856	Palaearctic (Trans-Palaearctic)	Temperate	herb, shrub
Dictynidae	*Emblyna mitis* (Thorell, 1875)	Euro-Siberian (European)	Temperate	herb
Gnaphosidae	*Drassyllus pusillus* (C.L.Koch, 1833)	Palaearctic (Trans-Palaearctic)	Temperate	ground, litter
Gnaphosidae	*Haplodrassus signifer* (C.L.Koch, 1839)	Holarctic (Circum-Holarctic)	Polyzonal	ground, litter
Gnaphosidae	*Micaria formicaria* (Sundevall, 1831)	Palaearctic (Trans-Palaearctic)	Subboreal	ground, litter
Gnaphosidae	*Micaria pulicaria* (Sundevall, 1831)	Holarctic (Circum-Holarctic)	Temperate	ground, litter
Gnaphosidae	*Micaria subopaca* Westring, 1861	Palaearctic (Trans-Palaearctic)	Temperate	ground, litter
Gnaphosidae	*Zelotes clivicola* (L.Koch, 1870)	Euro-Siberian (European-WestSiberian)	Temperate	ground, litter
Hahniidae	*Hahnia pusilla* C.L.Koch, 1841	Euro-Siberian (European-WestSiberian)	Temperate	litter
Linyphiidae	*Agyneta affinis* (KulczyE„ski, 1898)	Palaearctic (Trans-Palaearctic)	Temperate	litter
Linyphiidae	*Agyneta rurestris* (C.L.Koch, 1836)	Palaearctic (West-Palaearctic)	Polyzonal	litter
Linyphiidae	*Agyneta subtilis* (O.Pickard-Cambridge, 1863)	Palaearctic (West-Palaearctic)	Temperate	litter, moss
Linyphiidae	*Anguliphantes angulipalpis* (Westring, 1851)	Euro-Siberian (European-WestSiberian)	Temperate	litter
Linyphiidae	*Aphileta misera* (O.Pickard-Cambridge, 1882)	Holarctic (Circum-Holarctic)	Temperate	litter, moss

Linyphiidae	*Bathyphantes gracilis* (Blackwall, 1841)	Holarctic (Circum-Holarctic)	Polyzonal	litter, moss
Linyphiidae	*Bathyphantes nigrinus* (Westring, 1851)	Euro-Siberian (European-WestSiberian)	Temperate	litter, herb
Linyphiidae	*Bathyphantes parvulus* (Westring, 1851)	Palaearctic (Trans-Palaearctic)	Temperate	litter, moss
Linyphiidae	*Bolyphantes alticeps* (Sundevall, 1833)	Palaearctic (Trans-Palaearctic)	Temperate	herb
Linyphiidae	*Centromerita bicolor* (Blackwall, 1833)	Euro-Siberian (European)	Temperate	litter, moss
Linyphiidae	*Centromerita concinna* (Thorell, 1875)	Euro-Siberian (European)	subboreal	litter
Linyphiidae	*Ceratinella brevipes* (Westring, 1851)	Palaearctic (Trans-Palaearctic)	Temperate	litter
Linyphiidae	*Dicymbium nigrum* (Blackwall, 1834)	Palaearctic (West-CentralPalaearctic)	Temperate	litter
Linyphiidae	*Diplocephalus connatus* Bertkau, 1889	Euro-Siberian (European-MiddleSiberian)	Temperate	litter
Linyphiidae	*Diplocephalus cristatus* (Blackwall, 1833)	Palaearctic (TransPalaearctic)	Subboreal	litter
Linyphiidae	*Diplocephalus picinus* (Blackwall, 1841)	Palaearctic (West-Palaearctic)	Temperate	litter
Linyphiidae	*Diplostyla concolor* (Wider, 1834)	Holarctic (Circum-Holarctic)	Temperate	litter
Linyphiidae	*Drapetisca socialis* (Sundevall, 1833)	Palaearctic (Trans-Palaearctic)	Temperate	tree stems
Linyphiidae	*Enoplognatha ovata* (Clerck, 1757)	Holarctic (Circum-Holarctic)	Polyzonal	herb, shrub
Linyphiidae	*Entelecara acuminata* (Wider, 1834)	Palaearctic (West-Central-Palaearctic)	Subboreal	shrub, canopy
Linyphiidae	*Entelecara congenera* (O.Pickard-Cambridge, 1879)	Euro-Siberian (European-MiddleSiberian)	Boreal-mountain	canopy
Linyphiidae	*Erigone atra* Blackwall, 1833	Holarctic (Circum-Holarctic)	Polyzonal	ground, herb
Linyphiidae	*Erigone dentipalpis* (Wider, 1834)	Palaearctic (Trans-Palaearctic)	Polyzonal	ground, herb
Linyphiidae	*Erigonella hiemalis* (Blackwall, 1841)	Euro-Siberian (European-WestSiberian)	Temperate	litter, herb
Linyphiidae	*Gnathonarium dentatum* (Wider, 1834)	Palaearctic (Trans-Palaearctic)	Temperate	herb
Linyphiidae	*Gongylidium rufipes* (Linnaeus, 1758)	Palaearctic (Trans-Palaearctic)	Temperate	ground, herb
Linyphiidae	*Kaestneria pullata* (O.Pickard-Cambridge, 1863)	Holarctic (Circum-Holarctic)	Temperate	herb, shrub, canopy
Linyphiidae	*Lepthyphantes leprosus* (Ohlert, 1865)	Holarctic (Circum-Holarctic)	Temperate	tree stems, synanthrope
Linyphiidae	*Megalepthyphantes pseudocollinus* Saaristo, 1997	Palaearctic (West-Palaearctic)	Subboreal	shrub, tree stems

Linyphiidae	*Micrargus herbigradus* (Blackwall, 1854)	Palaearctic (Trans-Palaearctic)	Temperate	litter
Linyphiidae	*Micrargus subaequalis* (Westring, 1851)	Palaearctic (Trans-Palaearctic)	Temperate	litter
Linyphiidae	*Microlinyphia pusilla* (Sundevall, 1830)	Holarctic (Circum-Holarctic)	Polyzonal	herb
Linyphiidae	*Microneta viaria* (Blackwall, 1841)	Holarctic (Circum-Holarctic)	Temperate	litter
Linyphiidae	*Mioxena blanda* (Simon, 1884)	Euro-Siberian (European)	subboreal	litter
Linyphiidae	*Neriene clathrata* (Sundevall, 1830)	Palaearctic (Trans-Palaearctic)	Temperate	herb, shrub
Linyphiidae	*Nusoncus nasutus* (Schenkel, 1925)	Euro-Siberian (European)	subboreal	tree stems, herbs
Linyphiidae	*Oedothorax apicatus* (Blackwall, 1850)	Palaearctic (West-Central-Palaearctic)	Polyzonal	ground, litter
Linyphiidae	*Ostearius melanopygius* (O.Pickard-Cambridge, 1880)	Cosmopolitan (Cosmopolitan)	Synanthropic	ground
Linyphiidae	*Porrhomma microphthalmum* (O.Pickard-Cambridge, 1871)	Palaearctic (West-Central-Palaearctic)	Subboreal	litter
Linyphiidae	*Porrhomma pallidum* Jackson, 1913	Palaearctic (TransPalaearctic)	Temperate	litter
Linyphiidae	*Porrhomma pygmaeum* (Blackwall, 1834)	Palaearctic (Trans-Palaearctic)	Temperate	litter
Linyphiidae	*Praestigia kulczynskii* Eskov, 1979	Holarctic (Sub-CircumHolarctic)	Temperate	litter, moss
Linyphiidae	*Silometopus elegans* (O.Pickard-Cambridge, 1873)	Euro-Siberian (European-WestSiberian)	Subboreal	litter
Linyphiidae	*Silometopus reussi* (Thorell, 1871)	Palaearctic (Trans-Palaearctic)	Temperate	litter
Linyphiidae	*Tallusia experta* (O.Pickard-Cambridge, 1871)	Euro-Siberian (Trans-EuroSiberian)	Temperate	litter, moss
Linyphiidae	*Tapinocyba biscissa* (O.Pickard-Cambridge, 1873)	Euro-Siberian (European)	Subboreal	litter
Linyphiidae	*Tapinocyba insecta* (L.Koch, 1869)	Euro-Siberian (European-WestSiberian)	Temperate	litter
Linyphiidae	*Tenuiphantes mengei* (KulczyE„ski, 1887)	Palaearctic (Trans-Palaearctic)	Temperate	litter
Linyphiidae	*Tenuiphantes nigriventris* (L.Koch, 1879)	Euro-Siberian (NorthEuropean-Siberian)	Boreal-mountain	litter
Linyphiidae	*Tibioplus diversus* (L. Koch, 1879)	Holarctic (TransEuropeanSiberian-WestNearctic)	Boreal-mountain	litter
Linyphiidae	*Troxochrus scabriculus* (Westring, 1851)	Palaearctic (West-Palaearctic)	Subboreal	litter
Linyphiidae	*Walckenaeria antica* (Wider, 1834)	Palaearctic (Trans-Palaearctic)	Temperate	litter
Linyphiidae	*Walckenaeria dysderoides* (Wider, 1834)	Palaearctic (Trans-Palaearctic)	Temperate	litter, moss

Linyphiidae	*Walckenaeria nudipalpis* (Westring, 1851)	Euro-Siberian (Trans-EuroSiberian)	Temperate	litter, moss
Linyphiidae	*Walckenaeria unicornis* O.Pickard-Cambridge, 1861	Euro-Siberian (European-WestSiberian)	Temperate	Litter
Lycosidae	*Alopecosa pulverulenta* (Clerck, 1757)	Palaearctic (Trans-Palaearctic)	Temperate	ground
Lycosidae	*Pardosa agrestis* (Westring, 1861)	Palaearctic (West-Palaearctic)	Polyzonal	ground
Lycosidae	*Pardosa amentata* (Clerck, 1757)	Palaearctic (West-Palaearctic)	Polyzonal	ground
Lycosidae	*Pardosa fulvipes* (Collett, 1876)	Euro-Siberian (European-WestSiberian)	Temperate	ground
Lycosidae	*Pardosa lugubris* (Walckenaer, 1802)	Palaearctic (West-Palaearctic)	Temperate	ground
Lycosidae	*Pardosa paludicola* (Clerck, 1757)	Palaearctic (West-Palaearctic)	Subboreal	ground
Lycosidae	*Pardosa palustris* (Linnaeus, 1758)	Holarctic (Circum-Holarctic)	Polyzonal	ground
Lycosidae	*Pardosa plumipes* (Thorell, 1875)	Euro-Siberian (NorthEuropean-Siberian)	Polyzonal	ground
Lycosidae	*Pardosa prativaga* (L.Koch, 1870)	Palaearctic (West-Central-Palaearctic)	Temperate	ground
Lycosidae	*Pardosa sphagnicola* (Dahl, 1908)	Palaearctic (European-WestSiberian)	Boreal	ground
Lycosidae	*Pirata piraticus* (Clerck, 1757)	Holarctic (Circum-Holarctic)	Polyzonal	moss, aquatic
Lycosidae	*Piratula hygrophila* (Thorell, 1872)	Palaearctic (West-Central-Palaearctic)	Temperate	ground, moss
Lycosidae	*Trochosa ruricola* (De Geer, 1778)	Palaearctic (Trans-Palaearctic)	Polyzonal	ground
Lycosidae	*Xerolycosa miniata* (C.L.Koch, 1834)	Palaearctic (West-Central-Palaearctic)	Subboreal	ground
Mimetidae	*Ero cambridgei* KulczyE„ski, 1911	Palaearctic (TransPalaearctic)	Temperate	herbs shrub
Philodromidae	*Philodromus cespitum* (Walckenaer, 1802)	Holarctic (Circum-Holarctic)	Polyzonal	herb, shrub, canopy
Philodromidae	*Philodromus vinokurovi* Marusik, 1991	Euro-Siberian (EastEuropean-MiddleSiberian)	subboreal	Herb
Philodromidae	*Thanatus striatus* C. L. Koch, 1845	Holarctic (Circum-Holarctic)	Polyzonal	ground
Phrurolithidae	*Phrurolithus festivus* (C.L.Koch, 1835)	Palaearctic (Trans-Palaearctic)	Subboreal	ground, litter
Salticidae	*Euophrys frontalis* (Walckenaer, 1802)	Palaearctic (Trans-Palaearctic)	Temperate	litter, herb
Salticidae	*Evarcha arcuata* (Clerck, 1757)	Palaearctic (Trans-Palaearctic)	Temperate	herb
Salticidae	*Evarcha falcata* (Clerck, 1757)	Palaearctic (West-Central-Palaearctic)	Temperate	herb

Tetragnathidae	*Pachygnatha degeeri* Sundevall, 1830	Palaearctic (Trans-Palaearctic)	Polyzonal	ground, herb
Tetragnathidae	*Pachygnatha listeri* Sundevall, 1830	Euro-Siberian (Trans-EuroSiberian)	Temperate	ground, herb
Tetragnathidae	*Tetragnatha pinicola* L. Koch, 1870	Palaearctic (Trans-Palaearctic)	Polyzonal	herb, shrub
Theridiidae	*Coleosoma floridanum* Banks, 1900	Cosmopolitan (Cosmopolitan)	Synanthropic	Herb, ground
Theridiidae	*Episinus angulatus* (Blackwall, 1836)	AncientMediterranean (European-CentralAsian)	Subboreal	shrub
Theridiidae	*Episinus truncatus* Latreille, 1809	Palaearctic (West-Palaearctic)	Subboreal	shrub
Theridiidae	*Euryopis flavomaculata* (C.L.Koch, 1836)	Palaearctic (Trans-Palaearctic)	Temperate	litter
Theridiidae	*Howaiia mogera* (Yaginuma, 1972)	Cosmopolitan (Cosmopolitan)	Synanthropic	Litter, ground, herbs
Theridiidae	*Neottiura bimaculata* (Linnaeus, 1767)	Holarctic (Circum-Holarctic)	Temperate	herb
Theridiidae	*Parasteatoda tabulata* (Levi, 1980)	Cosmopolitan (Cosmopolitan)	Synanthropic	synanthrope
Theridiidae	*Phylloneta impressa* (L.Koch, 1881)	Holarctic (Circum-Holarctic)	Polyzonal	herb, shrub
Theridiidae	*Robertus arundineti* (O.Pickard-Cambridge, 1871)	Palaearctic (West-Central-Palaearctic)	Temperate	litter
Theridiidae	*Robertus neglectus* (O.Pickard-Cambridge, 1871)	Holarctic (Greenland-WestSiberian)	Temperate	litter, moss
Theridiidae	*Steatoda bipunctata* (Linnaeus, 1758)	Palaearctic (Trans-Palaearctic)	Temperate	tree stems, synanthrope
Theridiidae	*Steatoda castanea* (Clerck, 1757)	Palaearctic (Trans-Palaearctic)	Temperate	synanthrope
Theridiidae	*Steatoda grossa* (C.L.Koch, 1838)	Cosmopolitan (Cosmopolitan)	Synanthropic	synanthrope
Theridiidae	*Theridion mystaceum* L. Koch, 1870	Palaearctic (West-Central-Palaearctic)	Subboreal	herb, shrub, synanthrope
Thomisidae	*Ozyptila praticola* (C.L.Koch, 1837)	Palaearctic (West-Central-Palaearctic)	Temperate	litter
Thomisidae	*Xysticus cristatus* (Clerck, 1757)	Palaearctic (Trans-Palaearctic)	Polyzonal	herb
Thomisidae	*Xysticus kochi* Thorell, 1872	Palaearctic (West-Palaearctic)	Subboreal-semidesert	ground, herb
